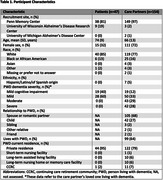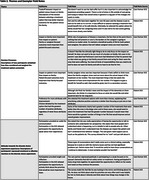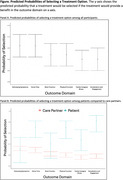# What matters most? A discrete choice experiment assessing preferences for dementia treatment outcomes

**DOI:** 10.1002/alz70858_107618

**Published:** 2025-12-26

**Authors:** Maayra I Butt, Carolyn Chow, Paola Rosa, Josiah Drakes, Melanie Bahti, Casey Whitman, Annalise Rahman‐Filipiak, Allison M Randall, Lindsay R Clark, Raghuram Iyengar, Kristin Harkins, Jason Karlawish, Scott D Halpern, Catherine L Auriemma

**Affiliations:** ^1^ Palliative and Advanced Illness Research Center, Perelman School of Medicine at the University of Pennsylvania, Philadelphia, PA, USA; ^2^ Department of Psychology, University of Nevada, Las Vegas, Las Vegas, NV, USA; ^3^ Department of Medicine, Perelman School of Medicine at the University of Pennsylvania, Philadelphia, PA, USA; ^4^ Michigan Alzheimer's Disease Research Center, Ann Arbor, MI, USA; ^5^ Wisconsin Alzheimer's Disease Research Center, University of Wisconsin School of Medicine and Public Health, Madison, WI, USA; ^6^ The Wharton School, University of Pennsylvania, Philadelphia, PA, USA; ^7^ Penn Memory Center, Perelman School of Medicine at the University of Pennsylvania, Philadelphia, PA, USA; ^8^ Leonard Davis Institute of Health Economics, Perelman School of Medicine at the University of Pennsylvania, Philadelphia, PA, USA

## Abstract

**Background:**

Persons living with dementia (PWD) and their family care partners may have different priorities when considering new dementia treatments. We sought to determine the relative importance of several different outcome domains when considering choosing a hypothetical dementia treatment.

**Method:**

We conducted a discrete choice experiment (DCE) among PWD and family care partners administered online and facilitated by a trained research coordinator. Participants were recruited from memory centers in Pennsylvania, Wisconsin, and Michigan. In each DCE task, participants were asked to choose one of two hypothetical dementia treatments that would produce variable benefits across multiple outcome domains. The full DCE, conducted with care partners, consisted of eight forced‐choice tasks assessing respondent preferences within six attributes: brain function, distressing symptoms, physical function, home time, family caregiver stress, and socialization and engagement. Participating PWD were shown an abbreviated version of the DCE consisting of six tasks with a random subset of four of the six attributes. Data were analyzed using mixed effects logistic regression models to assess how each attribute influenced likelihood of selecting a treatment option. Participant comprehension, reasoning, and reactions to the DCE were solicited throughout and documented in field notes. Field notes were subsequently analyzed using constant comparison techniques to identify relevant themes.

**Result:**

We surveyed 201 participants (154 care partners; 47 PWD; Table 1). Across all participants, the highest priority outcomes were relief in distressing symptoms and increasing home time (OR=0.66, 95% CI=0.61‐0.71 and OR=0.66, 95% CI=0.61‐0.71). When comparing utilities across PWD and care partners, PWD more highly valued increasing home time, improving physical function, reducing family caregiver stress, and increasing patient socialization and engagement compared to family care partners (Figure). Qualitative analysis revealed key themes regarding participant decision making processes and attitudes towards the DCE experience (Table 2).

**Conclusion:**

Increasing home time is an important treatment outcome for PWD and family care partners. Prioritization of other treatment outcomes vary across these key stakeholder subgroups. Participation in a DCE assessing treatment outcomes as a communication process was valued by patients and care partners, suggesting its potential utility as a communication aid or values elicitation tool.